# An Experimental Measurement Method to Characterize and Apply Platinum Silicon Material for a Biomechanical Replica of the Thoracic Aorta

**DOI:** 10.3390/biomimetics11040275

**Published:** 2026-04-16

**Authors:** Mario Alberto Grave-Capistrán, Francesco Lamonaca, Giuseppe Carbone, Christopher René Torres-SanMiguel

**Affiliations:** 1 Instituto Politécnico Nacional, Escuela Superior de Ingeniería Mecánica y Eléctrica, Sección de Estudios de Posgrado e Investigación, Unidad Zacatenco, Mexico City 07738, Mexico; 2 Department of Computer Science, Modelling, Electronic and System Science, University of Calabria, 87036 Rende, Italy; 3 Department of Mechanical, Energy and Management Engineering, University of Calabria, 87036 Rende, Italy

**Keywords:** platinum silicone, uncertainty, mechanical characterization, DIC method, biomechanical application, thoracic aorta

## Abstract

Currently, silicone is a common material used in medical research and biomedical applications. This research aims to characterize extra-soft platinum silicone (shore A 00 50) and compare its mechanical behavior with that of the human thoracic aorta. By developing molds to get samples, for tensile testing according to ISO 37 and ASTM D412, and for compression testing according to ISO 7743 and ASTM D575, using a universal testing machine for tensile and compression tests, and applying digital image correlation (DIC) algorithms, the mechanical properties were characterized in a total of 10 tensile samples and 6 compression samples. The results show an ultimate tensile strength up to 1.77 ± 0.12 MPa in the ASTM samples and 2.10 ± 0.14 MPa in the ISO samples; alongside an incremental elastic module of 80.08 ± 7.94 kPa and 117.98 ± 11.39 kPa; finally, an elongation at break of 1114.49 ± 76.77% and 936.08 ± 63.56%, corresponding to the values of a healthy thoracic aorta. The replica of the thoracic aorta in this material was developed by a brush method, with a thickness of 1.82 mm, a length from the aortic arch to the descending aorta of 200.49 mm, and diameters of 20.45 and 16.05 mm for the ascending and descending aorta, respectively.

## 1. Introduction

The biological tissues of the human body exhibit hyperelasticity, nonlinearity, and viscoelasticity. According to the literature on the mechanical properties of human arteries, the aorta has the highest value. The incremental elastic modulus ranges from 1000 kPa to 2000 kPa, and the ultimate tensile strength (UTS) can range from 800 kPa to 2180 kPa (20 to 60 years), with age being an important factor [1,2,3]. The current guidelines for establishing mechanical properties in materials like thermoset rubber and plastic elastomers are ASTM D412 (16th, 2021) and ISO 37 (7th, 2024) for tensile test [4,5], the standards for defining the mechanical properties in the same type of materials for compression tests are ASTM D575 (2024) and ISO 7743 (2017) [6,7]. Some research focuses on the mechanical characterization and development of silicone samples for tensile and compression testing, following standards that specify methodologies and materials [8,9]. Silicone is a polymeric material that is odorless and colorless, made primarily from silicon. It is flexible, resistant, functional, and able to withstand extreme temperatures. Silicone elastomers are generally considered to be physiologically inert and can be used for molding. Platinum silicone is a specific type of silicone made with platinum as a catalyst, making it a higher-quality material than standard silicone, with lower deformation and shrinkage. Its behavior shows a strong association with that of human arterial walls; it is appropriate to compare this material with the arterial walls due to their shared parameters, including tensile strength and viscoelastic properties [10,11,12]. Some research uses materials that mimic the behavior of the arterial wall, such as rubber, silicones, and elastomers; the mechanical properties and characteristics of each determine their similarity to biological tissue. Various investigations and industrial manufacturers have already developed replicas of the thoracic aorta by using silicones and flexible materials, or by applying 3D printing methods to flexible materials. Due to the geometry of the artery, some authors developed replicas by joining parts with silicone glue or by considering a specific region of the thoracic aorta; the thickness and mechanical properties depend on the material and method used to develop the replica [13,14,15,16,17,18,19,20,21,22].

This research aims to define the mechanical properties of the platinum silicone Ecoflex™ 00-50 through a methodology that has been specifically designed to evaluate the mechanical behavior of the material in uniaxial mechanical tests (tensile and compression) following the ASTM D412 and ASTM D575-91, as well as ISO 37 and ISO 7743 standards, developing molds for the samples using Fused Deposition Modeling (FDM), and in particular characterizing the incremental elastic modulus, the elongation at break (in percentage), and the tensile strength through a Universal Testing Machine. By comparing the results with the mechanical properties of human arteries in circumferential and longitudinal directions, this research builds on the application of platinum silicones for biomechanical analysis and on the development of a replica of the thoracic aorta.

## 2. Design Parameters

The mechanical properties are estimated by conducting experimental tests following a procedure to evaluate the elasticity of the materials. Uniaxial tests are performed on samples of the materials to be analyzed as part of the procedure; These samples exhibit characteristics and geometric shapes. The experimental tests are carried out using a universal uniaxial or biaxial tensile/compression testing machine, depending on the case study and interest. The experimental data allow for the definition of the material’s elastic and plastic behavior, as well as its peak stress. This information provides the stress and strain behavior curve of the material, the ultimate tensile strength (*UTS*), the elastic modulus (*E*), and the elongation. Young’s modulus, which measures the elastic behavior of a material, is calculated by dividing stresses (σ) by strains (ε) (in units of Pa) as the mechanical characterization of its elastic behavior. The following mathematical Expression (1) defines this parameter.

(1) $$E=\frac{\sigma }{\varepsilon }$$

Non-linear materials such as elastomers or rubber have a value of *E* that is determined by the strain; stress and strain changes in a small part of the stress–strain curve determine the incremental elastic modulus in accordance with Equation (2), where Δσ: increased stress between two load states; Δε: increased strain in the same two states; $\sigma_{n}$: stress values (initial and final); $\varepsilon_{n}$: strain values (initial and final).

(2) $$E_{inc}=\frac{\Delta \sigma }{\Delta \varepsilon }\;\to E_{inc}=\frac{\sigma_{2}-\sigma_{1}}{\varepsilon_{2}-\varepsilon_{1}}$$

The UTS of a material is the maximum stress it can withstand before failure. Equation (3) defines the maximum stress before the material fails divided by the material’s cross-sectional area [23].

(3) $$\sigma_{max}=\frac{F_{max}}{Cross-sectional\;area}$$

In compression testing of silicones, fracture usually does not occur due to the material’s properties. The compressive strength of these materials can be obtained at a specific deformation of the sample’s original height. The Poisson ratio of the material (v) allows for the calculation of the ratio of unit pressure change to corresponding volume change per unit of volume, which expresses the bulk modulus (K), which is the expression of compressibility. Equation (4) defines this [24].

(4) $$K=\frac{E}{3(1-2v)}$$

To process the results, it is necessary to verify their accuracy and quantify the associated margin of error, ensuring that the values are meaningful [25]. The next equation follows the expression to quantify the uncertainty in each measure:

(5) $$u\left(y\right)=\sqrt{{\left(\frac{\partial y}{{\partial x}_{1}}\cdot u\left(x_{1}\right)\right)}^{2}+{\left(\frac{\partial y}{{\partial x}_{2}}\cdot u\left(x_{2}\right)\right)}^{2}+\;\ldots \;{\left(\frac{\partial y}{{\partial x}_{n}}\cdot u\left(x_{n}\right)\right)}^{2}}$$

Platinum silicone was selected for its similarities to the arterial wall’s features and behavior, particularly regarding mechanical properties. Smooth-On Inc., Macungie, PA, USA, has developed Ecoflex™ platinum silicone 00-50, which has similar mechanical properties, such as a tensile strength up to 315 psi (as stated in technical documentation). Furthermore, they are materials with a low viscosity of 8000 cps; once the curing process is finished they develop a soft, resistant, and elastic consistency. In addition they can stretch up to 10 times their original size without breaking and can quickly return to their original shape without distortion [26]. The most common methods used for the application of platinum silicones are by injection or by mixing reagents (liquid state), which allow them to be molded into specific shapes, assisting the replication of geometries. Conducting uniaxial tests on the material using a specific procedure for characterization with samples is considered the appropriate procedure for validating technical information.

**Figure 1:**
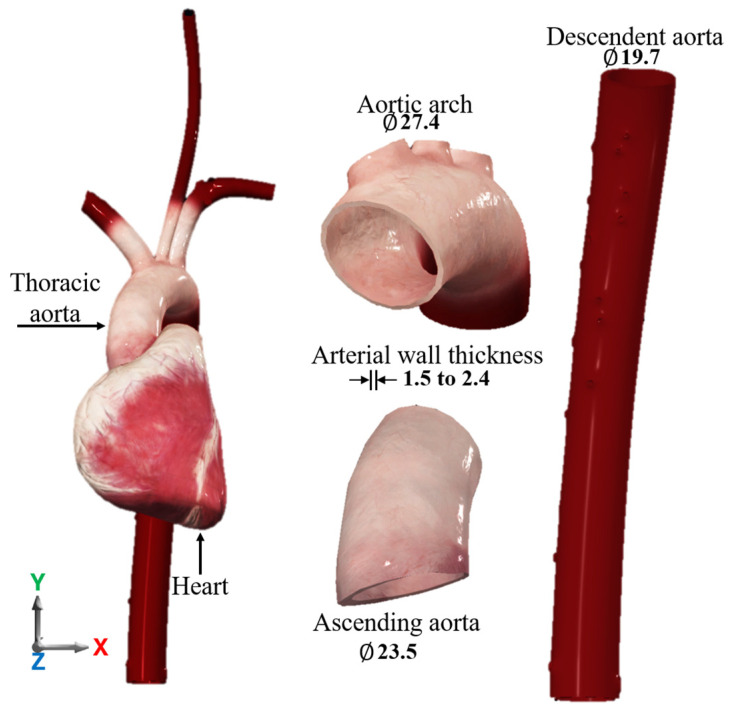
Dimensions (in mm) [28]. Frontal view of the thoracic aorta and its main three parts.

The thoracic aorta is a soft tissue belonging to the family of elastic fibers, located in the torso of the human body. It behaves as a hyperelastic, viscoelastic material that tolerates high stress and recovers its original shape. The thoracic aorta consists of three main parts: The ascending aorta, which has its origin in the left ventricle of the heart and where the aortic valve is located; the arch of the aorta, the central part of the thoracic aorta that constitutes the continuation of the artery and from which the supra aortic trunks originate and join with the arterial ligament of the left pulmonary artery; and the descending aorta, whose direction is towards the abdominal aorta. The mechanical behavior of the aortic wall has been described in various mathematical models; the constitutive models of Holzapfel, Gasser, and Ogden have been widely used, as they describe the behavior of healthy soft tissues [27]. Figure 1 shows the frontal view of the thoracic aorta, and the average of the dimensions in its main parts.

Based on the methodology of [29], a 3D mold will be developed to obtain a specific patient geometry of the thoracic aorta.

### 2.1. Samples Preparation

#### 2.1.1. Tensile Samples

The specifications for dumbbell-shaped specimens (types C and 1) are found in both standards, ASTM D412 and ISO 37, for uniaxial tensile tests. The main differences between them are the width of the narrow portion and the thickness of the sample. These standards are suitable for different elastomer evaluations, as they offer a wider range of testing procedures at different speeds (100–500 mm/min). Taking both standards into consideration, 3D models of the sample geometries (types C and 1) were created in the computer program SolidWorks^®^ student version (2017). Table 1 provides comprehensive information about the dimensions of both samples.

#### 2.1.2. Compression Samples

ASTM D575-91 and ISO 7743 propose cylindrical geometry for uniaxial compression tests. According to method “A”, the only difference between the geometries is the diameter of the cylindrical shape, which differs by 0.40 mm. Both standards propose an initial conditional cycle followed by the measurement cycle. For ASTM, there are 2 conditional cycles; for ISO, 3. Table 2 shows detailed information about the dimensions of these samples.

#### 2.1.3. Molding

**Figure 2:**
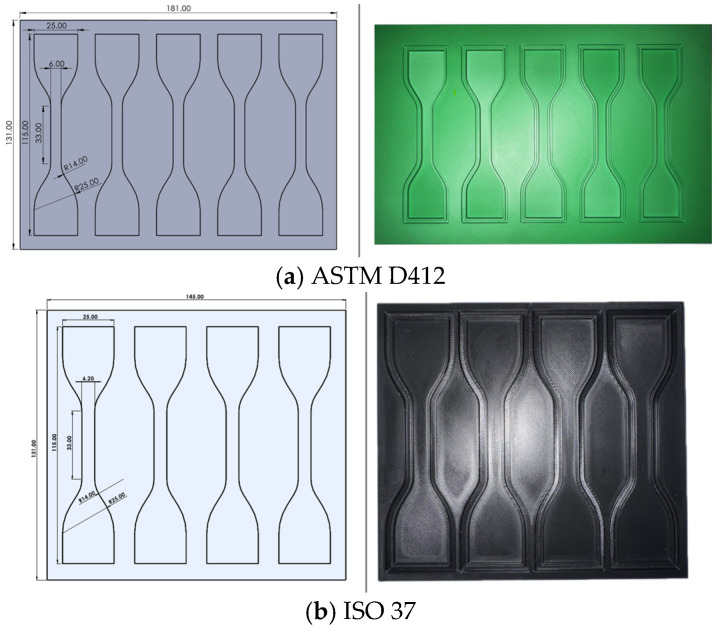

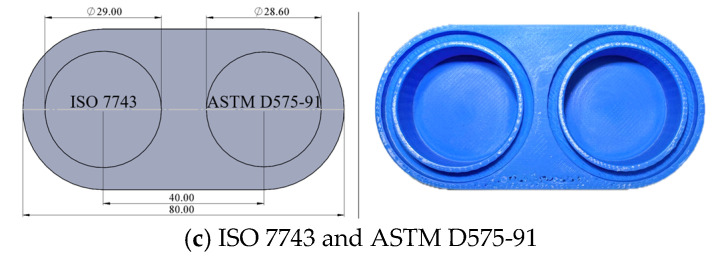
Dimensions (in mm) of the CAD tensile and compression molds, and the 3D printing post-processed; (**a**) ASTM D412 standard (**b**) ISO 37 standard, (**c**) ISO 7743 and ASTM D575-91 standards.

Molds for the samples were created using the X1 Carbon Bambu Lab 3D printer, developed by Shenzhen Tuozhu Technology, Shenzhen, China, the molds were made from Polylactic Acid (PLA). The printing nozzle had a diameter of 0.4 mm. The molds were printed at a speed of 50 mm/s for the first layer, 105 mm/s for full-filling, and 200 mm/s for exterior and interior walls. The acceleration configuration was determined as follows: 500 mm/s^2^ for the primary layer, 3000 mm/s^2^ for full filling, and 2000 mm/s^2^ for the exterior walls of the piece. Each shape of the 3D models was contoured with a spacing of 1.5 mm from the sample geometry, a width of 2 mm, and a depth of 2 mm before printing. The excess material will be stored in this contour without affecting the sample’s shape or thickness as silicone is released into the area. The flatness of the molds was enhanced by a post-process that included sanding at 45° with 220 and 1000 grit sandpaper and applying a pigment-free paint as a coating to achieve a smooth surface and seal the porosity, while maintaining the samples’ dimensions and tolerances. Figure 2 exhibits the development of the template mold for dumbbell-shaped and cylindrical samples (3D), as well as the post-processed 3D printed component. The mold used for ASTM D412 has a thickness of 5 mm. Each shape-geometry has a depth of 3 ± 0.20 mm. The ISO 37 template-mold is 5 mm thick, and the depth of the shape-geometry is 2 ± 0.20 mm. Meanwhile, the thickness of the mold for the cylindrical samples (ISO 7743 and ASTM D575-91) is 16.50 mm, and the depth of each shape geometry is 12.50 ± 0.50 mm.

### 2.2. Measurement Setup

**Figure 3:**
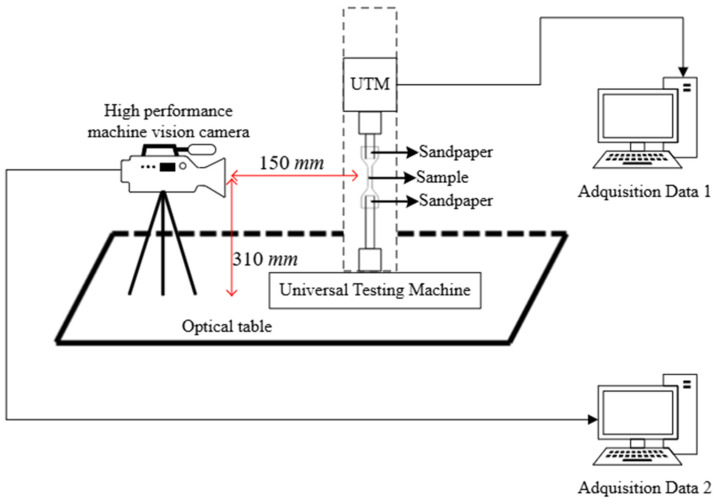
Test scenario: position of the machine vision camera and clamping in the universal testing machine during the tests.

Silicon samples were tested on the MTS Criterion Model 42 universal testing machine (MTS Systems Corporation, Eden Prairie, MN, USA), equipped with a load cell capable of 100 ± 0.5 N. The standards require the following configuration for uniaxial tensile tests: the clamps must maintain a constant clamping force while stretching the test piece at a specific speed, typically 100 ± 50 mm/min to 500 ± 50 mm/min, until the sample fails. The clamps held the sample couplings, including sandpaper with a porosity of 220, on both sides of the sample to improve anchorage and prevent the sample from slipping during the test; both types of samples were tested for tensile strength at 250 mm/min. On the other hand, the configuration for uniaxial compression tests involved changing the coupling system to parallel plates. The ISO essays consisted of 3 compression cycles at 10 mm/min, and in the fourth, the measure was obtained at 25% deflection. For the ASTM compression samples, two conditioning cycles were performed, and in the third cycle, the measurements were recorded at 12 mm/min and 25% deflection. Both parallel surfaces of the cylindrical samples were coated with oil for the experiments. Digital image correlation (DIC) was used to evaluate sample deformation during tensile testing. The DIC measurement system includes a GigE high performance machine vision camera (Prosilica GT, Allied Vision Technologies GmbH, Stadtroda, Germany), a 2/3 inch CCD sensor (Sony ICX625, Sony Corporation, Tokyo, Japan), characterized by a pixel size equal to 3.45 μm, a maximum frame rate of 15 fps and a resolution of 2448 × 2050 pixels at 80 mm in the lens. Image acquisition was carried out using the commercial software Vic-Snap 8 (Correlated Solutions Inc., Irmo, SC, USA). The VIC-2D software package (Correlated Solutions, Inc., USA, version 7) was used to analyze the speckle images. The stress–strain diagrams, Young’s modulus, UTS, and elongation at break were determined through mechanical tests and DIC measurements. Figure 3 shows the experimental setup of the universal testing machine and the position of the high-performance machine vision device.

**Figure 4:**
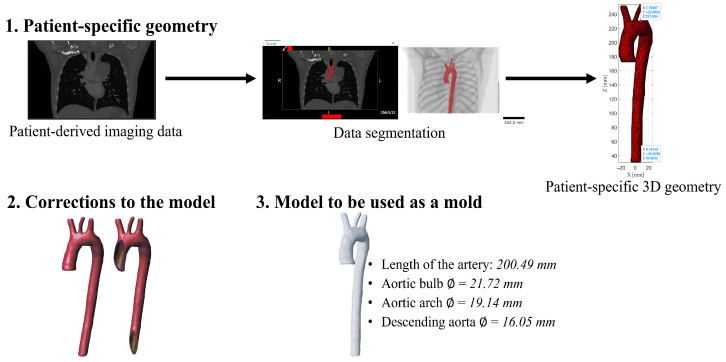
Methodology process to develop a specific patient geometry and mold for 3D printing.

The specific geometry modeling of the thoracic aorta was carried out using the MATLAB^®^ R2023a numerical computing system for academic use (Mathworks^®^, Natick, MA, USA) and a DICOM^®^ (295 images) file of the thorax of a healthy adult male at the 50th percentile. The procedure for creating three-dimensional modeling through the DICOM Browser tool belonging to the “Image Processing and Computer Vision” applications section, consists of reconstructing the thoracic aorta from the medical file through the segmentation of information corresponding to the artery, The next step is to convert the segmentation mask to a triangulated surface, defined by faces and vertices, then proceed to transform the triangulation into a file in STL format containing the specified geometry without alterations. Thus, modeling is analyzed in the Meshmixer^®^ (v3.5) software to correct surface imperfections. Finally, print the model to use as a mold. Figure 4 shows the methodology developed for this research to get mold from a specific patient geometry.

## 3. Experimental Setup

**Figure 5:**
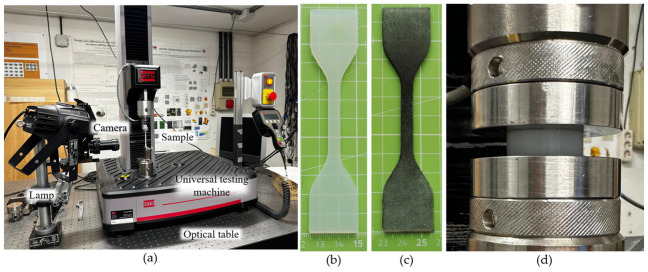
(**a**) Experimental scenario for the tensile and compression tests, (**b**) tensile sample (**c**) pigmented tensile sample for the DIC analysis, and (**d**) compression sample mounted.

Mixing silicone agents “A” (silicone elastomer base) and “B” (crosslinking component) at a 1:1 ratio (60 ± 2 mL of each) resulted in the production of 16 dumbbell-shaped samples. First, for the ASTM samples, the mixture (120 ± 4 mL) is gently mixed for 2 min at room temperature (26 °C) in a rectangular container (109 mm length × 70 mm height × 25 mm width), to prevent trapped air and excess bubbles, a lemniscate pattern was applied to the mixture, and to completely expel the trapped bubbles, a degassing procedure was performed using a two-stage vacuum pump and chamber. The degasification took 5 min and 28 s. After this step, the mixture was poured into the sample molds, and 8 ASTM samples were obtained according to the previous methodology. For the ISO samples, the same procedure was applied at room temperature (25 °C). A mixture of 120 ± 4 mL was used, and this time the degasification took a total of 9 min and 34 s. The mixture was poured into the ISO mold, obtaining eight samples. After 4 h of curing at room temperature (25 °C), the samples are ready for tensile testing. The sample surface was modified to produce a random speckle pattern that enables displacement field measurements. The high-resolution camera has been positioned in front of the samples at 150 mm and a height of 310 mm above the optical table. In addition, an LED light was used to improve the camera’s performance in capturing the pattern in the samples, positioned just behind the camera. The test scenario of the mechanical tests, the experimental setup (a), the tensile samples (b), (c) and compression sample mounted (d) are shown in Figure 5.

## 4. Experimental Evaluation

**Figure 6:**
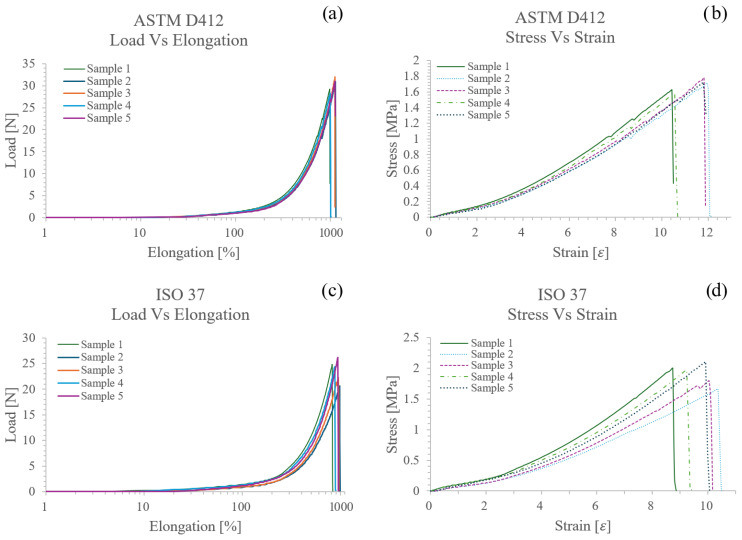
(**a**) ASTM D412 load-elongation graph; (**b**) ASTM D412 stress–strain graph; (**c**) ISO 37 load-elongation graph; (**d**) ISO 37 stress–strain graph, of platinum silicone samples.

Mechanical testing and mathematical analyses were used to determine the stress–strain diagram, incremental elastic modulus, UTS, and elongation at break. Figure 6a,c shows the graph of the results obtained from the data file generated by the universal testing machine (load and elongation). The load and cross-sectional area of the samples, 18 mm^2^ for ASTM D412 and 12.4 mm^2^ for ISO 37, can be utilized to calculate the UTS according to Equation (3) and the data. By defining a relationship between stress and strain, the maximum stress and strain can be expressed at the material’s breaking point. Figure 6b,d shows the highest levels of maximum stress and strain from its initial length (33 mm).

Additionally, to calculate the incremental elastic modulus of the material, two points in the linear range of the stress–strain curve were selected, and Equation (2) was used. The results are summarized in Table 3.

**Figure 7:**
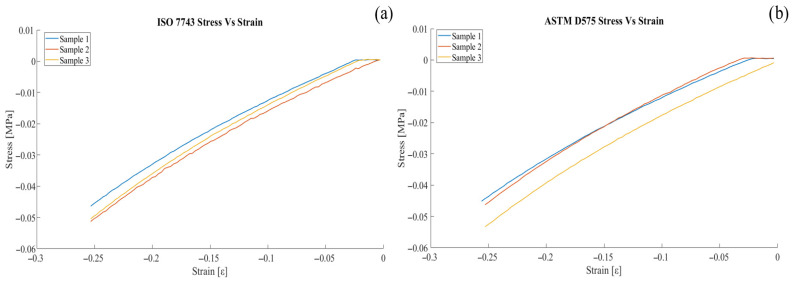
(**a**) Stress–strain behavior of ISO samples, and (**b**) stress–strain behavior of ASTM samples.

The stress–strain diagram in compression tests at 25% of deformation of the material is obtained by considering the cross-sectional area of the cylindrical samples. For ASTM samples, it is approximately. 642.42 mm, and for ISO samples, it is approximately 660.51 mm. Figure 7 shows the compressive behavior from the original height of samples (12.50 mm), (a) ISO 7743 samples, and (b) ASTM D575-91 samples.

Considering Equations (1) and (4), the stress–strain curve for compression tests at 25% deformation of the material is obtained. In addition, the bulk modulus is calculated using a Poisson ratio of 0.48 [30]. Table 4 shows the results of the Young’s modulus and Bulk modulus for each sample.

According to Equation (5) and factorizing, the uncertainty of the UTS can be defined as follows:

(6) $$u\left(UTS\right)=UTS\cdot \sqrt{{\left(\frac{{u(F}_{max})}{F_{max}}\right)}^{2}+{\left(\frac{{u(A}_{ini})}{A_{ini}}\right)}^{2}}$$

where ${u(F}_{max})$: Uncertainty of maximum stress (0.5 N); $F_{max}$: Maximum stress obtained (per sample); ${u(A}_{ini})$: Uncertainty of the initial cross-sectional area of the sample; $A_{ini}$: The initial cross-sectional area of the ASTM D test piece is 18 mm^2^, and for the ISO test piece is 12.4 mm^2^. To obtain the value of ${u(A}_{ini})$, Equation (5) is used considering the base and height of the cross section of the sample, as follows:

(7) $$u(A_{ini})=A_{ini}\cdot \sqrt{{\left(\frac{u\left(b\right)}{b}\right)}^{2}+{\left(\frac{u\left(h\right)}{h}\right)}^{2}}$$

where b: 6 mm and 6.2 mm; h: 3 mm and 2 mm; u(b): 0.01 mm; u(h): 0.01 mm. By substituting the values in Equation (7), the value in ASTM samples for ${u(A}_{ini})$ is 0.067, and the value in the ISO samples ${u(A}_{ini})$ is 0.065, usable for all corresponding samples. From the previous results and by substituting into Equation (6), the uncertainty for each sample is obtained; see Table 5.

**Figure 8:**
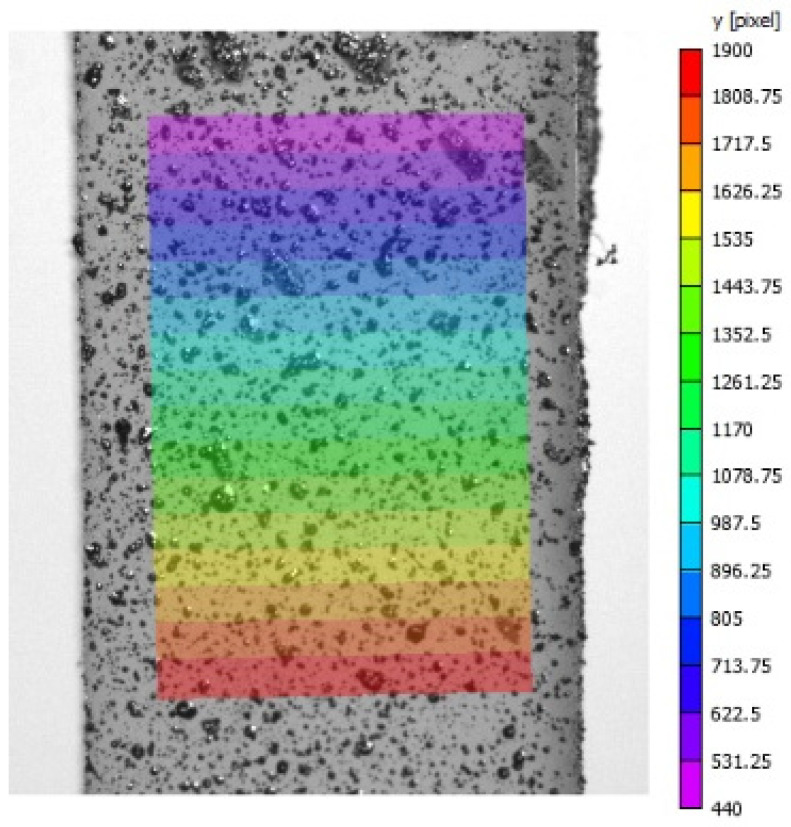
DIC Outcomes in the samples.

In addition, the displacement field obtained using the DIC method was analyzed and is reported in Figure 8.

**Figure 9:**
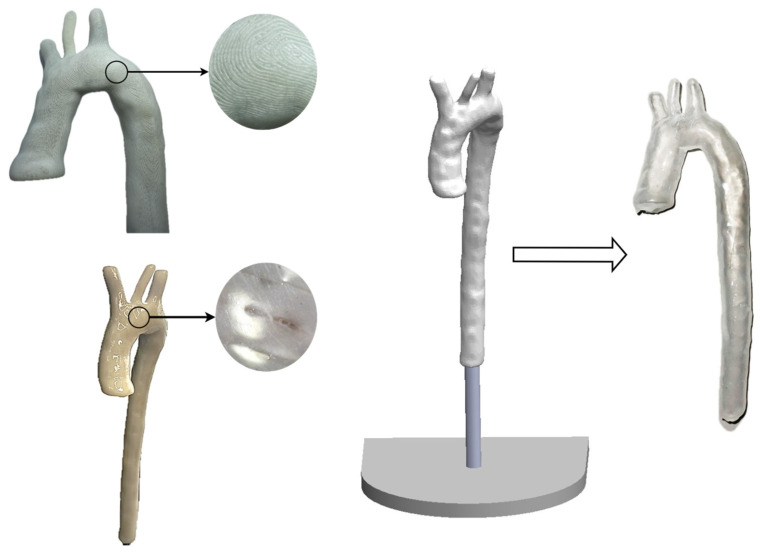
Mold printed in ABS; post-processing for the surface; and the brush method to create a replica of the thoracic aorta.

Irregularities were found on the surface of the model due to the printing layers after printing the patient-specific thoracic aortic geometry. To ensure a uniform surface with no irregularities or holes, a post-process was performed by sanding the 3D piece using the same methodology and applying a special coating. The mold was remade using a brush method to create a single piece of the artery due to the versatility of platinum silicone, 120 ± 6 mL (60 mL ± 1 mL for part A and 60 mL ± 1 mL for part B) of the material ECOFLEX 0050 was employed to cover a total of 3 layers. After the curing process, performed by sliding and releasing the cured material, a replica of the thoracic aorta was developed. Recirculating fluid and maintaining it within the replica was used to conduct a leakage test. The test lasted 5 min, and there was no fluid leakage. Figure 9 presents the 3D-printed, post-processed model, the brush method, and the replica of the thoracic aorta.

## 5. Discussion

Various studies on the characterization of aortic soft tissue have established that mechanical properties depend primarily on a person’s age and health. Different age groups have value definitions available (Avril, S. et al.). The mechanical characteristics of arteries, which include rupture and tensile strength (in axial and circumferential directions), are explored in research conducted in 2018. This research is based on an average rupture strength of 1.5 MPa in a healthy thoracic aorta [31]. Another work by Li, Z. et al. (2023) addressed the mean physiological modulus and the average elastic modulus of elastic fibers in proximal segments were assessed through uniaxial tensile tests on 50 samples of different age groups, the values are from 271 kPa to 440 kPa with a UTS (circumferential) up to 1.511 ± 0.62 MPa; the elastic modulus for distal segments ranges from 356 kPa to 386 kPa, and there is a UTS (circumferential) of up to 1.350 ± 0.50 MPa. The UTS reached its highest value in the youngest group (between 27 and 35 years) up to 1.892 ± 0.55 MPa [32]. According to the experimental results, the UTS of platinum silicon Ecoflex™ 0050 at 250 mm/min is up to 1.776 ± 0.12 MPa (ASTM D412), corresponding to the other standard, it is up to 2.107 ± 0.143 MPa (ISO 37); the material broke at a maximum elongation of 1114.48 ± 68.03% and 936.077%, respectively. The summarized results are shown in Table 6.

According to the results of Table 6 and the uncertainty for each sample, this material can be used as an approximation of the mechanical properties of a healthy aorta considering the UTS in the longitudinal and circumferential direction of the arterial wall in an age range between 35 and 55 [32] in addition to the fact that the material mimics structural behaviors due to mechanical loads.

## 6. Conclusions

The experimental evaluation of the platinum silicon material allows elastic behavior to be defined. Characterization of the platinum silicon Ecoflex™ 0050 using current standards enabled the determination of the UTS, elongation at break, incremental elastic modulus, and bulk modulus. Mathematical analysis and graphical results reveal the material’s behavior. According to the methodology used in this research, it is possible to define the mechanical properties of platinum silicones. According to the results, this material can be used to approximate the mechanical properties of a healthy aorta in both longitudinal and circumferential directions. Using this material to replicate arteries will enable structural biomechanical analysis in which the application of loads or pressure induces ruptures in the artery. In addition, this material can include fluid to analyze sudden occlusions or cardiac cycles. Finally, pathologies such as aneurysms can be analyzed by considering specific parameters such as the size and inflation of the artery wall.

## Figures and Tables

**Table 1:** Dimensions of the test piece (type 1 and type C) in the shape of a dumbbell.

**Description**	**Dimensions [mm]**		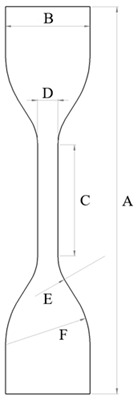
	**ISO “1”**	**ASTM “C”**	
A Total length	115	115	
B Width	25 ± 1.00	25 ± 1.00	
C Length of the measure section	33 ± 2.00	33 ± 2.00	
D Width of the measure section	6.2 ± 0.20	6 ± 0.05	
E Outer transition radius	14 ± 1.00	14 ± 1.00	
F Inner transition radius	25 ± 2.00	25 ± 2.00	
Gauge length	25 ± 0.50	25 ± 0.50	
Thickness of the sample	2 ± 0.20	3 ± 0.30	

**Table 2:** Dimensions of the test piece in the cylindrical shape.

**Description**	**Dimensions [mm]**		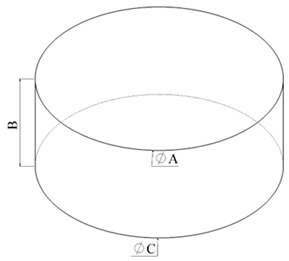
	**ISO “A”**	**ASTM “A”**	
A Diameter	29 ± 0.50	28.60 ± 0.10	
B Height	12.5 ± 0.50	12.5 ± 0.50	
C Diameter	29 ± 0.50	28.60 ± 0.10	

**Table 3:** Peak values of the UTS, $\varepsilon_{break}$ and $E_{inc}$.

Samples		UTS [MPa]	$\varepsilon_{break}$ [%]	$E_{inc}$ [kPa]
ASTM D412 Type C 250 mm/min	Sample 1	1.62	983.65	88.94
	Sample 2	1.71	1126.36	79.99
	Sample 3	1.77	1114.48	80.08
	Sample 4	1.57	995.55	87.55
	Sample 5	1.72	1111.01	72.87
ISO 37 Type 1 250 mm/min	Sample 1	1.97	817.15	113.92
	Sample 2	1.66	977.71	101.70
	Sample 3	1.79	947.98	96.28
	Sample 4	1.96	870.66	116.62
	Sample 5	2.10	936.07	117.98

**Table 4:** Compression results at 25% of the material’s deformation.

Samples		*E* [MPa]	Bulk Modulus [MPa]
ASTM D412 Type C 10 mm/min	Sample 1	0.18	1.50
	Sample 2	0.19	1.58
	Sample 3	0.20	1.67
ISO 37 Type 1 12 mm/min	Sample 1	0.18	1.50
	Sample 2	0.20	1.67
	Sample 3	0.20	1.67

**Table 5:** Absolute uncertainty values for each tensile sample.

Absolute Uncertainty				
Samples		UTS [MPa]	$\varepsilon_{break}$ [%]	$E_{inc}$ [kPa]
ASTM D412 Type C	Sample 1	±0.11	±68.10	±8.88
	Sample 2	±0.11	±77.73	±7.95
	Sample 3	±0.12	±76.76	±7.94
	Sample 4	±0.10	±69.06	±8.77
	Sample 5	±0.11	±76.65	±7.24
ISO 37 Type 1	Sample 1	±0.13	±55.73	±11.04
	Sample 2	±0.11	±67.97	±10.05
	Sample 3	±0.12	±65.32	±9.43
	Sample 4	±0.13	±59.45	±11.32
	Sample 5	±0.14	±63.55	±11.39

**Table 6:** Experimental results of Ecoflex™ 0050.

Samples		UTS [MPa]	$\varepsilon_{break}$ [%]	$E_{inc}$ [kPa]
ASTM D412 Type C 250 mm/min	Sample 1	1.62 ± 0.11	983.65 ± 68.10	88.94 ± 8.88
	Sample 2	1.71 ± 0.11	1126.36 ± 77.73	79.99 ± 7.95
	Sample 3	1.77 ± 0.12	1114.48 ± 76.76	80.08 ± 7.94
	Sample 4	1.57 ± 0.10	995.55 ± 69.06	87.55 ± 8.77
	Sample 5	1.72 ± 0.11	1111.01 ± 76.65	72.87 ± 7.24
ISO 37 Type 1 250 mm/min	Sample 1	1.97 ± 0.13	817.15 ± 55.73	113.92 ± 11.04
	Sample 2	1.66 ± 0.11	977.71 ± 67.97	101.70 ± 10.05
	Sample 3	1.79 ± 0.12	947.98 ± 65.32	96.28 ± 9.43
	Sample 4	1.96 ± 0.13	870.66 ± 59.45	116.62 ± 11.32
	Sample 5	2.10 ± 0.14	936.07 ± 63.55	117.98 ± 11.39

## Data Availability

Data is contained within the article.
